# Health vulnerability behaviors among men who have sex with men in the context of mpox: a cross-sectional study, Brazil, 2022

**DOI:** 10.1590/S2237-96222026v35e20250393.en

**Published:** 2026-07-20

**Authors:** Guilherme Reis de Santana Santos, Caíque Jordan Nunes Ribeiro, Thamyres Vaz de Jesus Bittencourt, Nilo Manoel Pereira Vieira Barreto, Lilian Conceição Guimarães Almeida, Tânia Christiane Ferreira Bispo, Virginia Ramos Santos Souza, Ana Paula Morais Fernandes, Anderson Reis de Sousa

**Affiliations:** 1 Universidade Federal de Sergipe Departamento de Enfermagem Lagarto SE Brazil Universidade Federal de Sergipe, Departamento de Enfermagem, Lagarto, SE, Brazil; 2 Universidade Federal da Bahia Escola de Enfermagem Salvador BA Brazil Universidade Federal da Bahia, Escola de Enfermagem, Salvador, BA, Brazil; 3 Universidade do Estado da Bahia Departamento de Ciências da Vida Salvador BA Brazil Universidade do Estado da Bahia, Departamento de Ciências da Vida, Salvador, BA, Brazil; 4 Universidade de São Paulo Escola de Enfermagem de Ribeirão Preto São Paulo SP Brazil Universidade de São Paulo, Escola de Enfermagem de Ribeirão Preto, São Paulo, SP, Brazil

**Keywords:** Sexual Vulnerability, Risk-Taking, Men’s Health, Mpox, Monkeypox, Cross-Sectional Studies, Vulnerabilidad sexual, Asunción de Riesgos, Salud del Hombre, Mpox, Estudios Transversales

## Abstract

**Objective:**

To analyze adherence to health vulnerability behaviors among men who have sex with men during the mpox public health emergency in Brazil.

**Method:**

A cross-sectional, analytical study conducted in a virtual environment between September and December 2022 via an electronic questionnaire, with recruitment via social media/dating apps. The outcome of interest comprised four variables related to health vulnerability behaviors, and a multivariable analysis was conducted using a generalized linear model.

**Results:**

A total of 1,449 men who reported having sex with men participated in the study, with a prevalence of health vulnerability behaviors of 13.9% (n=202), mainly due to not being vaccinated against mpox (50.4%), not adopting preventive care measures (30.6%), not undergoing testing (26.2%), and not following health treatment (9.9%). The multivariable model indicated a higher prevalence of the outcome independently associated with intimate contact with a person with mpox (adjusted prevalence ratio [APR]: 1.82; 95% confidence interval [95%CI] 1.33; 2.48), non-disclosure of serological status (APR 1.40; 95%CI 1.05; 1.87), and disbelief in mpox-related homophobia (APR 1.39; 95%CI 1.01; 1.90). The use of glory holes was associated with a lower prevalence (APR 0.43; 95%CI 0.20; 0.92).

**Conclusion:**

Greater health vulnerability was identified among participants who exhibited behaviors associated with a higher risk of mpox exposure, highlighting the need to plan targeted, population-based actions.

## Resumen

**Objetivo:**

Analizar la adhesión a comportamientos de vulnerabilidad en salud entre hombres que tienen sexo con hombres durante la emergencia de salud pública de mpox en Brasil.

**Método:**

Estudio transversal y analítico, realizado en un entorno virtual entre septiembre y diciembre de 2022 mediante un cuestionario electrónico, con reclutamiento en redes sociales y en aplicaciones de citas. El desenlace de interés del estudio estuvo compuesto por cuatro variables de comportamiento de vulnerabilidad en salud y se aplicó una regresión multivariada a los datos mediante el modelo de regresión lineal generalizado.

**Resultados:**

Participaron del estudio 1.449 hombres que afirmaron tener sexo con hombres, con una prevalencia de comportamientos de vulnerabilidad en salud del 13,9% (n=202), principalmente por no vacunarse contra la mpox (50,4%), no adoptar medidas de cuidado (30,6%), no realizar pruebas (26,2%) y no seguir tratamiento de salud (9,9%). El modelo multivariado indicó una mayor prevalencia del desenlace asociado de forma independiente al contacto íntimo con una persona con mpox (razones de prevalencia ajustadas [RPa]: 1,82; intervalos de confianza del 95% [IC95%] 1,33; 2,48), a no revelar el estado serológico (RPa 1,40; IC95% 1,05; 1,87) y a la falta de creencia en la homofobia relacionada con la mpox (RPa 1,39; IC95% 1,01; 1,90). El uso de glory holes (agujeros gloriosos) se asoció con una menor prevalencia (RPa 0,43; IC95% 0,20; 0,92).

**Conclusión:**

Se identificó una mayor vulnerabilidad en salud entre los participantes que presentaron comportamientos asociados a un mayor riesgo de exposición a la mpox, lo que requiere la planificación de acciones poblacionales específicas.

**Ethical aspects**

This research respected ethical principles, having obtained the following approval data:

Research ethics committee Universidade Federal da Bahia

Opinion number 6,065,498

Approval date 30/8/2022

Certificate of submission for ethical appraisal 61180222.8.0000.5531

Informed consent form Obtained from all participants prior to data collection.

## Introduction

The monkeypox virus, belonging to the genus Orthopoxvirus [1], gained notoriety following reports of human cases in endemic regions of West and Central Africa, particularly in the Democratic Republic of the Congo, where it remains endemic with sporadic outbreaks documented [2]. In May 2022, a confirmed case in the United Kingdom was followed by cases in several other previously non-endemic countries, demonstrating the virus’s rapid global spread [2].

Transmission to humans occurs through contact with bodily fluids from infected animals or humans [3]. In this context, mpox infection has gained epidemiological relevance due to global mobility, increased international travel, migratory flows, and interconnectivity among countries, all of which favor its spread [2].

Between January and December 2022, the World Health Organization (WHO) reported approximately 83,000 cases in 110 countries, of which 98% were among men with a mean age of 36 years. Of these, 95% were men who have sex with men, particularly in the 18 to 44-year age group, highlighting the vulnerability of this population [4]. Considering this scenario, WHO classified the 2022 outbreak as a Public Health Emergency of International Concern (PHEIC), exposing weaknesses in containment strategies, equitable access, and the provision of health care, as well as the impact of social stigma and barriers faced by vulnerable groups [4,5].

The characterization of the outbreak among men who have sex with men was marked by efforts in public health awareness actions and media communication, which ultimately reinforced prejudice and discrimination [5]. The response to mpox in this population, as in the HIV/AIDS pandemic, prompted reflections on society and health systems regarding epidemiological crises affecting historically marginalized groups [5]. 

In Brazil, between 2022 and July 2025, 14,458 mpox cases and 17 deaths were confirmed, with a peak in 2022 (10,840 cases), a sharp decline in 2023 (847 cases), a resurgence in 2024 (2,065), and a new decline in 2025 (706 by the end of that year). The burden is concentrated in the Southeast, particularly in the states of São Paulo and Rio de Janeiro, the latter leading, and, in 2025, the highest incidences occurred in Santa Catarina, Amazonas, and the Federal District. The profile of cases remains predominantly male and composed of young adults (mean age of 33 years). Among records with available information, sexual relations with men predominate, although there is a relevant proportion of missing data in variables related to sexual orientation and behavior [6].

Health vulnerability behaviors are attitudes, habits, or conditions that increase the risk of health problems and hinder the adoption of preventive practices and access to appropriate care. Such behaviors affect individuals’ health outcomes and, when linked to the social determinants of health, imply discrimination and stigma, highlighting the multifactorial nature of health-risk practices [7].

Stigma and discrimination may hinder access to health services, compromising the search for testing and early treatment [8]. Previous studies have suggested that the marginalization of men who have sex with men modifies the sexual behavior of this population, leading to an increase in unprotected sexual practices, multiple sexual partners, among other behaviors, which contributed to the spread of mpox during the 2022 outbreak [9,10]. The gap in educational and preventive campaigns, in turn, increases the risk of epidemic outbreaks in contexts of resource scarcity and low vaccination coverage, and delays decision-making by health authorities in providing care to vulnerable populations [11].

In 2024, Brazil implemented a vaccination coverage plan for populations at risk of mpox transmission as one of the measures to contain the disease [12]. However, adherence was hindered by unequal vaccine distribution, vaccine hesitancy, and limited information about vaccine safety [12]. In the same year, the resurgence of cases with the identification of clade Ib, a more infectious and potentially more severe variant than the 2022 mpox virus lineages, in the Democratic Republic of the Congo and neighboring countries, reinforced the need for more effective prevention and response strategies and the expansion of vaccination coverage [12,13]. 

Attention is drawn to the importance of strengthening health systems to address infectious diseases in a globalized world [14], a task that requires strategies that address discrimination and expand access to information and health services for key populations [8]. This study is justified by the specificity of mpox occurrence and care, shaped by structural failures in the public health system, social stigma, and barriers to access to services and to prevention policies, and by the need to develop effective and inclusive strategies to address epidemiological emergencies, with special attention to historically marginalized populations. 

In this context, the objective of this study was to analyze adherence to health vulnerability behaviors among men who have sex with men during the mpox public health emergency in Brazil.

## Methods

### Study design

This is a cross-sectional, analytical, and quantitative study conducted via a web survey, that is, an electronic questionnaire applied exclusively in a virtual environment. This manuscript followed the recommendations of the Strengthening the Reporting of Observational Studies in Epidemiology (STROBE).

### Setting

Data collection took place between September and December 2022, a period that coincided with the mpox outbreak in Brazil, during which reported cases were more concentrated in the states of São Paulo and Rio de Janeiro [15]. Participants were recruited exclusively in a virtual environment, through digital social networks (Facebook, Instagram, X), instant messaging applications (WhatsApp and Telegram), and dating and sexual networking applications (Hornet, Grindr, Scruff, and Tinder), all with a recognized queer and/or gay perspective. To enable participation, a specific research profile was created on these platforms, including a study description with a link to access the electronic questionnaire.

### Participants

The study population consisted of Brazilian men who used social networks and interaction applications. Inclusion criteria were: self-identifying as a man (cisgender or transgender); having had at least one sexual encounter with another man in the past 12 months; being 18 years of age or older; and residing in Brazil. Individuals who were outside the country during the data collection period were excluded from the analysis. Identification and recruitment of participants were conducted online through the dissemination of the research profile on social networks, instant messaging applications, and dating applications.

### Data sources and measurement

Data collection was carried out via an electronic questionnaire hosted on the REDCap platform. The instrument, validated (face and content) by five experts and five participants, consisted of 46 questions distributed across four sections: (a) sociodemographic information; (b) sexual-affective relationships; (c) sexual behaviors and practices; (d) use of health services, experiences, proximity, and perceptions regarding stigma/fear related to mpox.

Recruitment combined two strategies. First, the virtual snowball sampling technique [16] was used, in which 20 men who have sex with men, selected as “seeds” (diverse in terms of region, race/skin color, income, and education), were invited to share the research link within their networks. Second, direct dissemination was carried out through profiles created for the study on social networks (Facebook, Instagram, X), messaging applications (WhatsApp, Telegram), and dating applications (Hornet, Grindr, Scruff, and Tinder).

### Variables

The study outcome was a dichotomous variable (yes/no) composed of four behaviors: (1) would follow mpox treatment if diagnosed; (2) would undergo mpox testing if a testing center were available; (3) would receive vaccination against mpox, if the vaccine was available; (4) would take measures for self-care and notify contacts if diagnosed. If a participant responded “no” to at least one of the aforementioned variables, they were classified as “adherent to at least one health vulnerability behavior,” defined as at least one negative response regarding testing, treatment, vaccination, or contact notification related to mpox.

These behaviors were presented in the results of this manuscript, all measured dichotomously (yes/no): “Presented at least one vulnerability behavior”; “Presented two or more vulnerability behaviors”; “Would not be vaccinated against mpox”; “Would not take self-care/contact notification measures”; “Would not undergo mpox testing”; and “Would not follow treatment if diagnosed”. The first two variables were used exclusively for sample description.

Independent variables comprised three main dimensions:

Sociodemographic: being a young adult (<30 years) (yes/no); gender identity (cis/trans); homosexual orientation (yes/no); marital status (without a partner/with a partner); race/skin color (White/Non-White, including Black, Brown [Brazilian mixed race], Asian, and Indigenous, grouped according to recommendations [17]); having a religion (yes/no); higher education (yes/no); formal income (yes/no).
Psychosocial variables: previous diagnosis of mpox (yes/no); knowing people close who were diagnosed with mpox (yes/no); intimate contact with a person diagnosed with mpox (yes/no); would disclose mpox serological status (yes/no); fear of being discriminated against by health services (yes/no); fear of being discriminated against by friends (yes/no); fear of being discriminated against by family (yes/no); belief that someone may face homophobia because of mpox (yes/no).
Behavioral variables: type of sexual partnership (last six months) (no partner/fixed/fixed+casual); was immunized against COVID-19 (yes/no); stopped engaging in casual sex after the mpox outbreak (yes/no); would undergo pre- and post-exposure testing for mpox (yes/no); condomless sexual practice (yes/no); uses apps to seek sex (yes/no); uses saunas to seek sex (yes/no); seeks sex in public places (parties and clubs) (yes/no); use of glory hole (yes/no); practice of chemsex (yes/no); practice of cruising (yes/no); practice of bugchasing (yes/no); challenging sexual practices (yes/no); and participation in orgies (yes/no).

The following practices were defined based on previous studies [16,18]: (a) double penetration: simultaneous sexual penetration by two or more penises; (b) challenging sexual practice: consistent engagement in two or three of these practices, defined based on the circumstances in which they occur; (c) fisting or footing: anal penetration using the fist or foot; (d) cruising: free, consensual, and anonymous sex practiced between men in public spaces, such as parks, wooded areas, beaches, or parking lots; (e) glory hole: a round opening in a wall that allows insertion of the penis, so that someone on the other side can use it sexually in various ways; (f) bugchasing: when an HIV-negative man deliberately seeks an HIV-positive man to become infected. 

To define chemsex, participants were asked whether they had used illicit drugs immediately before and/or during sexual intercourse in the past 12 months. For those who answered yes, a multiple-choice list was presented to indicate the specific drugs used [18].

### Bias

To reduce selection bias, the virtual snowball technique was adopted, favoring the inclusion of participants from different regions, sociodemographic profiles, and sexual contexts. The questionnaire underwent face and content validation and pilot testing, which minimized measurement bias. Online data collection allowed for greater anonymity, reducing underreporting of stigmatized practices.

### Study size

Sample size calculation was performed using G*Power software, version 3.1.9.7, considering the population of men aged >18 years in Brazil. The assumed prevalence was 50% (to maximize sample size due to the lack of prior data on this phenomenon), with a tolerable standard error of 3% and a 95% confidence level. The estimated sample size at the end was 1,067 men.

### Statistical methods

Statistical analyses were conducted using IBM SPSS software (SPSS Inc., Chicago, IL, USA), version 27.0. An exploratory analysis was performed to describe the distribution of predictor variables in relation to health vulnerability behavior, with data presented as absolute and relative frequencies.

Pearson’s chi-square test was used to assess the association between predictor variables and adherence to health vulnerability behaviors and to screen candidate variables for the initial multivariable model. Variables with p-value<0.020 in the bivariate analysis were considered eligible to compose this model.

The presence of multicollinearity was investigated using tolerance coefficients and the variance inflation factor (VIF). The final stage of the analysis consisted of multivariable modeling to identify factors independently associated with health vulnerability behavior among men who have sex with men. The generalized linear Poisson regression model was applied with a log link function because the outcome prevalence was >10%. Adherence to the Poisson distribution was assessed using the Kolmogorov-Smirnov test (p-value>0.005) and the assumption of equidispersion. 

The calculation of adjusted prevalence ratios (APR) and 95% confidence intervals (95%CI) used the hybrid parameter estimation method, the robust variance estimator, and Type III analysis to test model effects. The omnibus test was used to evaluate the hypothesis of the final multivariable model, which explains factors associated with health vulnerability behavior, against a model containing only the intercept, at a significance level of 5% (p-value<0.005). 

The final model was adjusted for age and showed a good fit to the data (deviance 771.862; AIC 1.187.862; BIC 1.219.517; log-likelihood –587.931). The omnibus test indicated that the overall model was significant (χ^2^(5)=23.048; p-value<0.001). The significance of the APR for variables included in the final model was assessed using the Wald chi-square test, with variables having p-values<0.005 considered significant.

## Results

Among study participants, 13.9% (n=202) of the 1,449 men who reported having sex with men presented at least one health vulnerability behavior, while 86.1% (n=1,247) reported none of these behaviors. Among vulnerable participants, 17.3% (n=35/202) presented two or more vulnerability behaviors. Specifically, 50.4% (n=102/202) reported that they would not be vaccinated against mpox, 30.6% (n=62/202) stated that they would not take self-care/contact notification measures, 26.2% (n=53/202) that they would not undergo mpox testing, and 9.9% (n=20/202) reported that they would not follow treatment if diagnosed (Table 1).

**Table 1:** Distribution of health vulnerability behaviors among men who have sex with men in the context of mpox. Brazil, 2022 (n=1,449)

Variables	Yes n (%)	No n (%)	Vulnerable n=202 (%)
Presented at least one vulnerability behavior	202 (13.9)	1,247 (86.1)	-
Presented two or more vulnerability behaviors	35 (2.4)	1,414 (97.6)	17.3 (35)
Would not be vaccinated against mpox	102 (7.0)	1,347 (93.0)	102 (50.4)
Would not take self-care/contact notification measures	62 (4.3)	1,387 (95.7)	62 (30.6)
Would not undergo mpox testing	53 (3.7)	1,396 (96.3)	53 (26.2)
Would not follow treatment if diagnosed	20 (1.4)	1,429 (98.6)	20 (9.9)

Of the total study participants, young adults (<30 years) were identified (n=654; 45.1%), who were predominantly cisgender (n=1,400; 96.6%) and reported a homosexual orientation (n=1,222; 84.3%). Marital status without a partner was more frequent (n=1,094; 75.5%), as well as non-White race/skin color (n=867; 59.8%). Regarding religiosity, 49.5% (n=717) reported being religious. Higher education was reported by 88.1% (n=1,276) of participants, and formal income by 73.2% (n=1,061). No sociodemographic variable showed statistical significance with respect to vulnerability behaviors (p-value>0.005) (Table 2).

**Table 2:** Bivariate analysis of sociodemographic factors associated with health vulnerability behaviors among men who have sex with men in the context of mpox. Brazil, 2022 (n=1,449)

Variables		Health vulnerability behaviors			p-value^a^
		Yes (n=202)	No (n=1,247)	Total (n=1,449)	
		n (%)	n (%)	n (%)	
Young adult (<30 years)	Yes	84 (12.8)	570 (87.2)	654 (45.1)	0.274
	No	118 (14.8)	677 (85.2)	795 (54.9)	
Gender identity	Trans	3 (6.1)	46 (93.9)	49 (3.4)	0.108
	Cis	199 (14.2)	1,201 (85.8)	1.400 (96.6)	
Homosexual orientation	Yes	178 (14.6)	1,044 (85.4)	1.222 (84.3)	0.111
	No	24 (10.6)	203 (89.4)	227 (15.7)	
Marital status	Without a partner	151 (13.8)	943 (86.2)	1.094 (75.5)	0.761
	With a partner	51 (14.4)	302 (85.6)	353 (24.4)	
Race/skin color	Non-White	118 (13.6)	749 (86.4)	867 (59.8)	0.658
	White	84 (14.4)	498 (85.6)	582 (40.2)	
Religion	Yes	106 (14.8)	611 (85.2)	717 (49.5)	0.254
	No	91 (12.7)	625 (87.3)	716 (49.4)	
Higher education	Yes	177 (13.9)	1.099 (86.1)	1,276 (88.1)	0.836
	No	25 (14.5)	148 (85.5)	173 (11.9)	
Formal income	Yes	144 (13.6)	917 (86.4)	1,061 (73.2)	0.503
	No	58 (14.9)	330 (85.1)	388 (26.8)	

^a^Differences were considered statistically significant when p-value<0.005.

Among psychosocial factors related to prior experience with the mpox virus, 14.6% (n=211) of participants reported a previous mpox diagnosis. In comparison, 25.5% (n=369) reported knowing close individuals who had been diagnosed with mpox. Additionally, 12.1% (n=175) reported having had intimate contact with a person diagnosed with mpox (p-value<0.001), 78.6% (n=1,139) stated that they would disclose their mpox serological status, and non-disclosure was associated with greater vulnerability (p-value 0.095). Regarding social perceptions, fear of discrimination was reported in health services (n=810; 55.9%), among friends (n=948; 65.4%), and among family members (n=743; 51.3%). Finally, 84.1% (n=1,218) reported the belief that someone may face homophobia because of mpox, and the absence of this belief was associated with greater vulnerability (p-value 0.040) (Table 3).

**Table 3:** Bivariate analysis of psychosocial factors associated with health vulnerability behaviors among men who have sex with men in the context of mpox. Brazil, 2022 (n=1,449)

Variables		Health vulnerability behaviors			p-value^a^
		Yes (n=202)	No (n=1,247)	Total(n=1,449)	
		n (%)	n (%)	n (%)	
Previous diagnosis of mpox	Yes	43 (20.4)	168 (79.6)	211 (14.6)	0.003
	No	159 (12.8)	1,079 (87.2)	1,238 (85.4)	
Knows close individuals who have been diagnosed with mpox	Yes	52 (14.1)	317 (85.9)	369 (25.5)	0.922
	No	150 (13.9)	930 (86.1)	1,080 (74.5)	
Intimate contact with a person diagnosed with mpox	Yes	41 (23.4)	134 (76.6)	175 (12.1)	<0.001
	No	161 (12.6)	1,113 (87.4)	1,274 (87.9)	
Would disclose mpox serological status	Yes	150 (13.2)	989 (86.8)	1,139 (78.6)	0.095
	No	52 (16.9)	256 (83.1)	308 (21.3)	
Fear of discrimination by health services	Yes	123 (15.2)	687 (84.8)	810 (55.9)	0.142
	No	79 (12.5)	554 (87.5)	633 (43.7)	
Fear of discrimination by friends	Yes	133 (14.0)	815 (86.0)	948 (65.4)	0.893
	No	69 (13.8)	432 (86.2)	501 (34.6)	
Fear of discrimination by family	Yes	114 (15.3)	629 (84.7)	743 (51.3)	0.114
	No	88 (12.5)	618 (87.5)	706 (48.7)	
Belief that someone may face homophobia because of mpox	Yes	159 (13.1)	1,059 (86.9)	1,218 (84.1)	0.022
	No	43 (18.8)	186 (81.2)	229 (15.8)	

^a^Differences were considered statistically significant when p-value<0.005.

Among behaviors, 67.0% (n=971) reported that the type of sexual partnership (last six months) was fixed+casual, and 99.9% (n=1,447) stated that they had been immunized against COVID-19. Regarding changes after the outbreak, 55.1% (n=799) reported that they stopped engaging in casual sex after the mpox crisis. Regarding testing, 83.9% (n=1,216) reported that they would undergo pre- and post-exposure testing for mpox (Table 4). 

**Table 4:** Bivariate analysis of behavioral factors associated with health vulnerability behaviors among men who have sex with men in the context of mpox. Brazil, 2022 (n=1,449)

Variables		Health vulnerabilityn behaviors			p-value^a^
Yes (n=202)	No (n=1,247)	Total (n=1,449)
n (%)	n (%)	n (%)
Type of sexual partnership (last six months)	No partner	10 (11.2)	79 (88.8)	89 (6.1)	0.419
	Fixed	59 (15.6)	318 (84.4)	377 (26.0)	
	Fixed + Casual	131 (13.5)	840 (86.5)	971 (67.0)	
Was vaccinated against COVID-19	Yes	201 (13.9)	1,246 (86.1)	1,447 (99.9)	0.141
	No	1 (50.0)	1 (50.0)	2 (0.1)	
Stopped engaging in casual sex after the mpox outbreak	Yes	106 (13.3)	693 (86.7)	799 (55.1)	0.411
	No	96 (14.8)	554 (85.2)	650 (44.9)	
Would undergo pre- and post-exposure testing for mpox	Yes	129 (10.6)	1,087 (89.4)	1,216 (83.9)	<0.001
	No	42 (38.5)	67 (61.5)	109 (7.5)	
Condomless sexual practice	Yes	105 (13.0)	703 (87.0)	808 (55.8)	0.243
	No	97 (15.1)	544 (84.9)	641 (44.2)	
Uses apps to seek sex	Yes	112 (13.6)	709 (86.4)	821 (56.7)	0.707
	No	90 (14.3)	538 (85.7)	628 (43.3)	
Uses saunas to seek sex	Yes	23 (10.6)	194 (89.4)	217 (15.0)	0.123
	No	179 (14.5)	1,053 (85.5)	1,232 (85.0)	
Seeks sex in public places (parties and clubs)	Yes	50 (11.0)	403 (89.0)	453 (31.3)	0.031
	No	152 (15.3)	844 (84.7)	996 (68.7)	
Use of glory hole	Yes	7 (7.4)	88 (92.6)	95 (6.6)	0.056
	No	195 (14.4)	1,159 (85.6)	1,354 (93.4)	
Practice of chemsex	Yes	58 (20.6)	224 (79.4)	282 (19.5)	<0.001
	No	144 (12.3)	1,023 (87.7)	1,167 (80.5)	
Practice of cruising	Yes	27 (14.3)	162 (85.7)	189 (13.0)	0.883
	No	175 (13.9)	1,085 (86.1)	1,260 (87.0)	
Practice of bugchasing	Yes	32 (25.4)	94 (74.6)	126 (8.7)	<0.001
	No	170 (12.8)	1,153 (87.2)	1,323 (91.3)	
Challenging sexual practices	Yes	46 (20.5)	178 (79.5)	224 (15.5)	0.002
	No	156 (12.7)	1,069 (87.3)	1,225 (84.5)	
Participation in orgies	Yes	24 (14.9)	137 (85.1)	161 (11.1)	0.707
	No	178 (13.8)	1,110 (86.2)	1,288 (88.9)	

^a^Differences were considered statistically significant when p-value<0.005.

In terms of sexual practices, 55.8% (n=808) reported condomless sex, 56.7% (n=821) reported using apps to seek sex, 15.0% (n=217) reported using saunas to seek sex, and 31.3% (n=453) reported seeking sex in public places (parties and clubs). Regarding specific practices, the use of glory holes (n=95; 6.6%) showed a tendency toward being associated with a lower prevalence of the outcome, although without statistical significance (p-value 0.056). The practice of chemsex (n=282; 19.5%) showed a significant association (p-value<0.001), while the practice of cruising (n=189; 13.0%) did not show statistical significance (p-value 0.883). The practices of bugchasing (n=126; 8.7%) (p-value<0.001) and challenging sexual practices (n=224; 15.5%) (p-value 0.002) were also associated with the outcome. Participation in orgies (n=161; 11.1%) was not statistically significant (p-value 0.707) (Table 4).

The multivariable model included an adjustment variable and identified three variables associated with a higher prevalence of health vulnerability behaviors: having intimate contact with someone diagnosed with mpox (APR 1.82; 95%CI 1.33; 2.48); not disclosing mpox serological status (APR 1.40; 95%CI 1.05; 1.87); and not believing that people may face homophobia due to mpox (APR 1.39; 95%CI 1.01; 1.90). The use of glory holes for sexual activity was associated with a 57% lower prevalence of the outcome (APR 0.43; 95%CI 0.20; 0.92) (Table 5).

## Discussion

This study identified that social and behavioral factors strongly influenced the adoption of health vulnerability behaviors among men who have sex with men during the mpox outbreak in Brazil. Having intimate contact with individuals diagnosed with mpox, not disclosing serological status, and not recognizing the possibility of homophobia-related discrimination were associated with higher vulnerability, whereas the use of alternative sexual practices, such as glory holes, emerged as a protective factor. These findings showed that risk perception and the ways individuals negotiate intimacy directly impact preventive health behaviors.

The study had some limitations. The sample included users of social networks/dating applications recruited via virtual snowball sampling, which may have limited participation of individuals with lower digital access and introduced selection bias. Data collection was self-reported and anonymous, which, although it ensures confidentiality and encourages participation, may introduce recall and social desirability biases. The cross-sectional design may prevent causal inference. The categorization of race/skin color into “White” and “Non-White,” due to the low number of Black and Indigenous individuals in the sample, may have limited intersectional analysis. This limitation highlights the importance of future studies that are racially representative and employ broader analytical approaches. The creation of a composite outcome was also a limitation, as it combined distinct behaviors with different underlying causes. Nevertheless, the study provided relevant evidence for understanding how social inequalities and perceptions of stigma shape health vulnerability.

In the multivariable model, intimate contact with a person diagnosed with mpox was the most strongly associated factor. Emotional closeness may reduce risk perception and lead to less cautious decision-making [19]. The possibility of denial or risk minimization among individuals exposed to the virus should be considered, indicating the need for post-exposure testing and contact monitoring strategies [20]. 

In situations where the virus is already present in an immediate social circle (e.g., a diagnosed individual), risk perception may be underestimated, leading to less cautious behavior [9] and a false sense of familiarity and trust among intimate partners, resulting in lower adherence to preventive measures [21]. In contexts of emotional/sexual closeness, there is a tendency to overlook basic precautions, highlighting the need for educational strategies focused on individuals close to diagnosed persons [21,22].

Non-disclosure of mpox serological status was relevant, as it was associated with a higher likelihood of health vulnerability behaviors, possibly linked to fear of discrimination/stigma and homophobia [23,24]. This silence, although socially protective, increases individual and collective vulnerability and disrupts transmission chains [25]. Anti-stigma actions to enable disclosure of serological status without social retaliation should be implemented [26]. 

The disbelief in the possibility of homophobia associated with mpox was associated with a higher likelihood of health vulnerability behaviors. This finding suggests that minimizing the impact of prejudice may lead to a lower perception of social risk and, consequently, to reduced preventive care [26]. The denial of discrimination risk indicates a distancing from the reality of experiencing prejudice, highlighting the need for educational campaigns that integrate both biological and social risk dimensions. It reinforces that perceived social risk (discrimination) influences health behaviors among men who have sex with men, underscoring the need for broader awareness campaigns that go beyond biological framing and include the social consequences of the disease and empathy [26,27]. 

On the other hand, using glory holes for sexual activity appeared protective. This may reflect a perceived reduction in direct contact, even though the practice still carries significant risks of transmitting infection. This finding highlights the complexity of self-protection strategies, which may simultaneously be perceived as protective and risky [28]. The use of glory holes may involve sexual activities with exposure to bodily fluids, representing a considerable risk for the spread of infections such as mpox [28]. 

There was a predominance of behaviors such as condomless sex (bareback), use of apps to find partners, and the presence of casual and steady partners. These behaviors can be identified as risk factors for the transmission of sexually transmitted infections, as shown in previous studies in Brazil [29]. However, some participants reported behavioral changes, such as stopping casual sexual encounters after the mpox outbreak, indicating opportunities for educational interventions [20,29].

In the context of mpox, health vulnerability among men who have sex with men is shaped by social and behavioral factors, ranging from intimate contact with diagnosed individuals to non-disclosure of serological status and underestimation of homophobia, as well as the rejection of alternative sexual strategies that may function as relative protection. Addressing this vulnerability, beyond individual-level behaviors among affected populations, requires integrating biomedical measures with stigma reduction, safe care environments, equitable public policies, qualified listening practices, and social justice.

## Data Availability

The database and analysis codes used in the research are available for consultation upon request by contacting the responsible research team.
