# Supplementary material for: Health vulnerability behaviors among men who have sex with men in the context of mpox: a cross-sectional study, Brazil, 2022
Source: Epidemiol Serv Saude. 2026 Jul 20;35:e20250393. doi: 10.1590/S2237-96222026v35e20250393.en (PMC13686301; doi:10.1590/S2237-96222026v35e20250393.en)
Supplement: Translation

# Translation: Comportamentos de vulnerabilidade à saúde entre homens que fazem sexo com homens no contexto da mpox: estudo transversal, Brasil, 2022

**DOI:** 10.1590/S2237-96222026v35e20250393.pt

**Authors:** Guilherme Reis de Santana Santos, Caíque Jordan Nunes Ribeiro, Thamyres Vaz de Jesus Bittencourt, Nilo Manoel Pereira Vieira Barreto, Lilian Conceição Guimarães Almeida, Tânia Christiane Ferreira Bispo, Virginia Ramos Santos Souza, Ana Paula Morais Fernandes, Anderson Reis de Sousa

**Affiliations:** 1 Universidade Federal de Sergipe Departamento de Enfermagem Lagarto SE Brasil Universidade Federal de Sergipe, Departamento de Enfermagem, Lagarto, SE, Brasil; 2 Universidade Federal da Bahia Escola de Enfermagem Salvador BA Brasil Universidade Federal da Bahia, Escola de Enfermagem, Salvador, BA, Brasil; 3 Universidade do Estado da Bahia Departamento de Ciências da Vida Salvador BA Brasil Universidade do Estado da Bahia, Departamento de Ciências da Vida, Salvador, BA, Brasil; 4 Universidade de São Paulo Escola de Enfermagem de Ribeirão Preto São Paulo SP Brasil Universidade de São Paulo, Escola de Enfermagem de Ribeirão Preto, São Paulo, SP, Brasil

**Keywords:** Vulnerabilidade Sexual, Assunção de Riscos, Saúde do Homem, Mpox, Estudos Transversais

## Abstract

**Objetivo:**

Analisar a adesão a comportamentos de vulnerabilidade à saúde entre homens que fazem sexo com homens na emergência de saúde pública de mpox no Brasil.

**Método:**

Estudo transversal, analítico, realizado em ambiente virtual entre setembro e dezembro de 2022 via questionário eletrônico, com recrutamento em redes sociais/aplicativos de relacionamento. O desfecho de interesse do estudo foi composto por quatro variáveis de comportamentos de vulnerabilidade à saúde e foi aplicada regressão multivariada dos dados pelo modelo de regressão linear generalizado.

**Resultados:**

Participaram do estudo 1.449 homens que afirmaram fazer sexo com homens, uma prevalência de comportamentos de vulnerabilidade à saúde de 13,9% (n=202), principalmente por não se vacinarem contra a mpox (50,4%), não adotarem medidas de cuidado (30,6%), não realizarem testagem (26,2%) e não seguirem tratamento de saúde (9,9%). O modelo multivariado indicou maior prevalência do desfecho associada independentemente a contato íntimo com pessoa com mpox (razões de prevalência ajustada [RP ajustada]: 1,82; intervalos de confiança de 95% [IC95%] 1,33; 2,48), a não revelação do status sorológico (RP ajustada 1,40; IC95% 1,05; 1,87) e à descrença em homofobia relacionada à mpox (RP ajustada 1,39; IC95% 1,01; 1,90). O uso de glory holes (buracos da glória) associou-se à menor prevalência (RP ajustada 0,43; IC95% 0,20; 0,92).

**Conclusão:**

Identificou-se maior vulnerabilidade à saúde entre participantes que apresentaram comportamentos relacionados ao maior risco de exposição ao mpox, o que enseja planejamento de ações populacionais específicas.

**Aspectos éticos**

Esta pesquisa respeitou os princípios éticos, obtendo os seguintes dados de aprovação:

Comitê de ética em pesquisa Universidade Federal da Bahia

Número do parecer 6.065.498

Data de aprovação 30/8/2022

Certificado de apresentação de apreciação ética 61180222.8.0000.5531

Registro do consentimento livre e esclarecido Obtido de todos os participantes antes da coleta.

## Introdução

O vírus monkeypox, pertencente ao gênero Orthopoxvirus [1], ganhou notoriedade após relatos de casos humanos em regiões endêmicas das regiões ocidental e central da África, particularmente na República Democrática do Congo, onde permanece endêmico com registros de surtos esporádicos [2]. Em maio de 2022, um caso confirmado no Reino Unido foi seguido por ocorrências em diversos outros países, até então não endêmicos, o que demonstrou rápida disseminação global do vírus [2].

A transmissão para humanos ocorre por contato com fluidos corporais de animais ou de humanos infectados [3]. Nesse contexto, a infecção por mpox ganha relevância epidemiológica devido à mobilidade global, ao aumento de viagens internacionais, ao fluxo migratório e à interconectividade entre os países, fatores que favorecem sua disseminação [2].

Entre janeiro e dezembro de 2022, a Organização Mundial da Saúde (OMS) notificou cerca de 83 mil casos em 110 países, dos quais 98% foram em homens com idade média de 36 anos. Destes, 95% correspondiam a homens que fazem sexo com homens, sobretudo na faixa etária 18 a 44 anos, o que evidenciou a vulnerabilidade desse grupo [4]. Com a consideração desse cenário, a OMS classificou o surto de 2022 como emergência de saúde pública de importância internacional, que expunha fragilidades nas estratégias de contenção, no acesso equitativo e na produção do cuidado em saúde, além de impacto do estigma social e das barreiras enfrentadas por grupos vulneráveis [4,5].

A caracterização do surto em homens que fazem sexo com homens foi marcada por esforços em ações de conscientização em saúde pública e em comunicação midiática, o que acabou por reforçar preconceitos e discriminações [5]. A resposta à mpox nesse público, como na pandemia de HIV/aids, despertou reflexões sobre a sociedade e os sistemas de saúde quanto às crises epidemiológicas em grupos historicamente marginalizados [5]. 

No Brasil, entre 2022 e julho de 2025, foram confirmados 14.458 casos de mpox e 17 óbitos, com pico em 2022 (10.840 casos), queda acentuada em 2023 (847 casos), recrudescimento em 2024 (2.065) e novo arrefecimento em 2025 (706 até o fim desse ano). A carga concentra-se no Sudeste, sobretudo nos estados de São Paulo e do Rio de Janeiro, este à frente, e, em 2025, as maiores incidências ocorreram em Santa Catarina, no Amazonas e no Distrito Federal. O perfil dos casos permanece majoritariamente masculino e de adultos jovens (média de 33 anos). Entre os registros com informação, predominam relações sexuais com homens, embora haja proporção relevante de dados ignorados nas variáveis de orientação e de comportamento [6].

Os comportamentos de vulnerabilidade à saúde são atitudes, hábitos ou condições que aumentam o risco de agravos à saúde, o que dificulta a adoção de práticas preventivas e o acesso a cuidados adequados. Tais condutas impactam os resultados de saúde dos indivíduos e, quando relacionadas aos determinantes sociais da saúde, implicam em discriminação e em estigma, o que evidencia a natureza multifatorial das práticas de risco em saúde [7].

O estigma e a discriminação podem dificultar o acesso aos serviços de saúde, o que compromete a busca por testagem e o tratamento precoce [8]. Estudos anteriores sugeriram que a marginalização dos homens que fazem sexo com homens modifica o comportamento sexual dessa população, o que leva ao aumento da incidência de práticas sexuais desprotegidas, de múltiplos parceiros sexuais, dentre outras práticas, o que contribuiu para a disseminação da mpox durante o surto em 2022 [9,10]. O hiato de campanhas educativas e preventivas, por sua vez, aumenta o risco de surto epidêmico em contextos de escassez de recursos e de baixa cobertura vacinal, além de retardar a tomada de decisão das autoridades sanitárias no sentido de oferecer atenção às populações vulneráveis [11].

Em 2024, o Brasil implementou planejamento de cobertura vacinal para populações de risco de contágio da mpox como uma das medidas para contenção da doença [12]. A adesão, entretanto, foi prejudicada por distribuição desigual de vacinas, hesitação vacinal e informações limitadas sobre a segurança do imunizante [12]. No mesmo ano, o recrudescimento de casos com a identificação do clado Ib, variante mais infecciosa e potencialmente mais grave que as linhagens de 2022 do vírus mpox, na República Democrática do Congo e em países vizinhos, reforçou a necessidade de estratégias mais efetivas de prevenção, enfrentamento e ampliação da cobertura vacinal [12,13]. 

Alerta-se sobre a importância do fortalecimento dos sistemas de saúde para o enfrentamento de doenças infecciosas em um mundo globalizado [14], o que demanda estratégias que considerem o combate à discriminação e a ampliação do acesso à informação e aos serviços de saúde para as populações-chave [8]. Este estudo justifica-se pela especificidade de acometimento e de atenção à mpox, circunstanciada em falhas estruturais do sistema público, em estigma social e em barreiras de acesso aos serviços e às políticas de prevenção, o que contribui para o desenvolvimento de estratégias eficazes e inclusivas de enfrentamento a emergências epidemiológicas, com especial atenção às populações historicamente marginalizadas. 

O objetivo deste estudo, nesse contexto, foi analisar a adesão a comportamentos de vulnerabilidade à saúde entre homens que fazem sexo com homens na emergência de saúde pública da mpox no Brasil.

## Métodos

### Delineamento do estudo

Trata-se de estudo transversal, analítico e quantitativo, realizado por meio de web-survey, ou seja, inquérito eletrônico aplicado exclusivamente em ambiente virtual. Este manuscrito seguiu as recomendações do *Strengthening the Reporting of Observational Studies in Epidemiology *(STROBE).

### Contexto

A coleta de dados ocorreu entre setembro e dezembro de 2022, período que coincidiu com o surto da mpox no Brasil, com maior concentração de casos notificados nos estados de São Paulo e do Rio de Janeiro [15]. O recrutamento dos participantes ocorreu exclusivamente em ambiente virtual, por meio de redes sociais digitais (Facebook, Instagram, X), aplicativos de mensagens instantâneas (WhatsApp e Telegram) e aplicativos de encontros românticos e sexuais (Hornet, Grindr, Scruff e Tinder), todos com reconhecida perspectiva *queer *e/ou gay. Para viabilizar a participação, foi criado um perfil específico da pesquisa nessas plataformas, com a descrição do estudo contendo link de acesso ao questionário eletrônico.

### Participantes

A população do estudo foi composta por homens brasileiros usuários de redes sociais e aplicativos de interação. Os critérios de inclusão foram: identificar-se como homem (cisgênero ou transgênero); ter tido pelo menos um encontro sexual com outro homem nos últimos 12 meses; ter 18 anos ou mais; e residir no Brasil. Foram excluídos da análise os indivíduos que, durante o período de coleta de dados, estiveram fora do país. A identificação e o recrutamento dos participantes ocorreram de forma on-line, a partir da divulgação do perfil da pesquisa em redes sociais, em aplicativos de mensagens instantâneas e em aplicativos de encontros.

### Fontes de dados e mensuração

A coleta de dados foi realizada por meio de questionário eletrônico hospedado na plataforma REDCap. O formulário, validado (face-conteúdo) por cinco especialistas e cinco participantes, possuía 46 questões distribuídas em quatro seções: (a) informações sociodemográficas; (b) relacionamentos sexuais-afetivos; (c) comportamentos e práticas sexuais; (d) utilização de serviços de saúde, vivências, proximidade e percepções sobre estigma/medo relacionado à mpox.

O recrutamento combinou duas estratégias. Na primeira, utilizou-se a técnica de amostragem *snowball virtual *[16], em que 20 homens que fazem sexo com homens, selecionados como “sementes” (diversificados quanto à região, raça/cor da pele, renda e escolaridade), foram convidados a compartilhar o link da pesquisa em suas redes. Na segunda, houve divulgação direta em perfis criados para o estudo em redes sociais (Facebook, Instagram, X), em aplicativos de mensagens (WhatsApp, Telegram) e em aplicativos de encontros (Hornet, Grindr, Scruff e Tinder).

### Variáveis

O desfecho do estudo foi uma variável dicotômica (sim/não) composta por quatro comportamentos: (1) seguiria o tratamento da mpox, caso fosse diagnosticado; (2) realizaria a testagem da mpox, caso houvesse centro de testagem disponível; (3) se vacinar contra a mpox, caso o imunizante estivesse disponível; (4) tomaria providências para se cuidar e comunicar às pessoas com que mantiveram contato, caso fosse diagnosticado. Caso o participante respondesse “não” a pelo menos uma das variáveis mencionadas, ele seria computado como “adepto a pelo menos um comportamento de vulnerabilidade em saúde”, o qual seria caracterizado como ao menos uma negativa entre testagem, tratamento, vacinação ou notificação de contatos frente à mpox.

Esses comportamentos foram apresentados nos resultados deste manuscrito, todos mensurados de forma dicotômica (sim/não): “Apresentou pelo menos um comportamento de vulnerabilidade”; “Apresentou dois ou mais comportamentos de vulnerabilidade”; “Não se vacinaria contra a mpox”; “Não tomaria medidas de autocuidado/notificação”; “Não realizaria testagem para a mpox”; e “Não seguiria tratamento em caso de diagnóstico”. As duas primeiras variáveis foram utilizadas exclusivamente para descrição da amostra.

As variáveis independentes contemplaram três dimensões principais:

Sociodemográficas: ser adulto jovem (<30 anos) (sim/não); identidade de gênero (cis/trans); orientação homossexual (sim/não); situação conjugal (sem um parceiro/com um parceiro); raça/cor de pele (branca/não branca, entre pretos, pardos, amarelos e indígenas, agrupados de acordo com recomendação [17]; possuir religião (sim/não); ensino superior (sim/não); renda formal (sim/não).
Variáveis psicossociais: diagnóstico anterior de mpox (sim/não); conhece pessoas próximas que foram diagnosticadas com mpox (sim/não); contato íntimo com uma pessoa diagnosticada com mpox (sim/não); divulgaria o estado sorológico da mpox (sim/não); medo de ser discriminado pelos serviços de saúde (sim/não); medo de ser discriminado pelos amigos (sim/não); medo de ser discriminado pela família (sim/não); crença de que alguém pode sofrer homofobia por causa da mpox (sim/não).
Variáveis comportamentais: tipo de parceria sexual (últimos seis meses) (sem parceiro/fixo/fixo+casual); foi imunizado contra a covid-19 (sim/não); parou de fazer sexo casual após o surto de mpox (sim/não); seria submetido a testes de pré e de pós-exposição para mpox (sim/não); prática sexual sem preservativo (sim/não); usa aplicativos para procurar sexo (sim/não); usa saunas para procurar sexo (sim/não); procura sexo em locais públicos (festas e clubes) (sim/não); uso do *glory hole* (sim/não); prática de chemsex (sim/não); prática de cruising (sim/não); prática de bugchasing (sim/não); práticas sexuais desafiadoras (sim/não); e participação em orgias (sim/não).

As seguintes práticas foram definidas com base em estudos anteriores [16,18]: (a) dupla penetração: penetração sexual simultânea por dois ou mais pênis; (b) prática sexual desafiadora: envolvimento consistente em duas ou três dessas práticas, definidas com base nas circunstâncias em que ocorrem; (c) fisting ou footing: penetração anal usando o punho ou o pé; (d) cruising: sexo livre, consensual e anônimo praticado entre homens em espaços públicos, como parques, matas, praias ou estacionamentos; (e) *glory hole*: abertura redonda em uma parede que permite a inserção do pênis, para que alguém do outro lado utilize-o sexualmente de variadas formas; (f) bugchasing: quando um homem HIV negativo procura deliberadamente um homem HIV positivo para ser infectado. 

Para definir chemsex, foi perguntado se os participantes consumiram drogas ilícitas imediatamente antes e/ou durante a relação sexual nos últimos 12 meses. Para aqueles que responderam afirmativamente, apresentamos uma lista de múltipla escolha para indicar os medicamentos específicos consumidos [18].

### Viés

Para reduzir o viés de seleção, adotou-se a técnica *snowball virtual* (bola de neve virtual), que favoreceu a inclusão de participantes de diferentes regiões, perfis sociodemográficos e contextos sexuais. O questionário passou por validação face-conteúdo e teste-piloto, o que minimizou o viés de mensuração. A coleta on-line permitiu maior anonimato, o que reduziu a subnotificação de práticas estigmatizadas.

### Tamanho do estudo

O cálculo amostral utilizou o software G*Power, versão 3.1.9.7, com a consideração da população de homens >18 anos no Brasil. A prevalência assumida foi 50% (para maximizar o tamanho da amostra, devido à falta de dados prévios sobre a prevalência desse fenômeno), com um erro padrão tolerável de 3% e um nível de confiança de 95%. A amostra estimada ao final foi de 1.067 homens.

### Métodos estatísticos

As análises estatísticas foram conduzidas com a utilização do software IBM SPSS (SPSS Inc., Chicago, IL, EUA), versão 27.0. Foi realizada análise exploratória para descrever a distribuição das variáveis preditoras em relação ao comportamento de vulnerabilidade em saúde, com apresentação dos dados em frequências absolutas e relativas.

O teste qui-quadrado de Pearson foi empregado para verificar a associação entre as variáveis preditoras e a adesão a comportamentos de vulnerabilidade em saúde, com a finalidade de triagem de variáveis candidatas ao modelo multivariável inicial. Foram consideradas elegíveis para compor esse modelo as variáveis com p-valor<0,020 na análise bivariada.

A presença de multicolinearidade foi investigada, com respeito aos parâmetros dos coeficientes de tolerância e do fator de inflação da variância (VIF, *variance inflation factor*). A etapa final da análise consistiu na modelagem multivariada para identificar os fatores independentemente associados ao comportamento de vulnerabilidade em saúde entre homens que fazem sexo com homens. O modelo de regressão linear generalizado de Poisson se deu com função de ligação logarítmica, já que a prevalência do desfecho foi >10%. A aderência à distribuição de Poisson considerou o teste de Kolmogorov-Smirnov (p-valor>0,005) e a suposição de equidispersão. 

O cálculo das razões de prevalência ajustada (RP ajustada) e dos intervalos de confiança de 95% (IC95%) utilizou o método híbrido de estimação de parâmetros, o estimador de variância robusta e a análise tipo III para testar os efeitos do modelo. O teste omnibus foi empregado para avaliar a hipótese do modelo multivariado final, a fim de explicar os fatores associados ao comportamento de vulnerabilidade em saúde em comparação a modelo contendo apenas o intercepto, com nível de significância de 5% (p-valor<0,005). 

O modelo final foi ajustado por idade e apresentou boa aderência aos dados (deviance 771,862; AIC 1.187,862; BIC 1.219,517; log-verossimilhança –587,931). O teste omnibus indicou significância do modelo geral (χ^2^(5)=23,048; p-valor<0,001). A significância das RP ajustadas das variáveis incluídas no modelo final foi analisada por meio do teste qui-quadrado de Wald, no que foram significativas aquelas com p-valor<0,005.

## Resultados

Entre os participantes do estudo, os 1.449 homens que afirmaram fazer sexo com homens, 13,9% (n=202) apresentaram pelo menos um comportamento de vulnerabilidade à saúde, enquanto 86,1% (n=1.247) não relataram nenhuma prática desse tipo. Dentre os participantes vulneráveis, 17,3% (n=35/202) apresentaram dois ou mais comportamentos de vulnerabilidade. Especificamente, 50,4% (n=102/202) declararam que não se vacinariam contra a mpox, 30,6% (n=62/202) afirmaram que não tomariam medidas de autocuidado/notificação, 26,2% (n=53/202) que não realizariam testagem para a mpox e 9,9% (n=20/202) relataram que não seguiriam tratamento em caso de diagnóstico (Tabela 1).

**Tabela 1:** Distribuição dos comportamentos de vulnerabilidade à saúde entre homens que fazem sexo com homens no contexto da mpox. Brasil, 2022 (n=1.449)

Variáveis	Sim n (%)	Não n (%)	Vulneráveis n=202 (%)
Apresentou pelo menos um comportamento de vulnerabilidade	202 (13,9)	1.247 (86,1)	
Apresentou dois ou mais comportamentos de vulnerabilidade	35 (2,4)	1.414 (97,6)	17,3 (35)
Não se vacinaria contra a mpox	102 (7,0)	1.347 (93,0)	102 (50,4)
Não tomaria medidas de autocuidado/notificação	62 (4,3)	1.387 (95,7)	62 (30,6)
Não realizaria testagem para a mpox	53 (3,7)	1.396 (96,3)	53 (26,2)
Não seguiria tratamento em caso de diagnóstico	20 (1,4)	1.429 (98,6)	20 (9,9)

Do total de participantes do estudo, foram identificados adultos jovens (<30 anos) (n=654; 45,1%), com identidade de gênero cis (n=1.400; 96,6%) e orientação homossexual (n=1.222; 84,3%). A situação conjugal sem um parceiro foi mais frequente (n=1.094; 75,5%), assim como a raça/cor da pele não branca (n=867; 59,8%). Sobre a religiosidade, 49,5% (n=717) afirmaram possuir religião. O ensino superior foi relatado por 88,1% (n=1.276) dos participantes e a renda formal por 73,2% (n=1.061). Nenhuma variável sociodemográfica mostrou significância estatística em relação aos comportamentos de vulnerabilidade (p-valor>0,005) (Tabela 2).

**Tabela 2:** Análise bivariada de fatores sociodemográficos associados aos comportamentos de vulnerabilidade à saúde entre homens que fazem sexo com homens no contexto da mpox. Brasil, 2022 (n=1.449)

Variáveis		Comportamentos de vulnerabilidade à saúde			p-valor^a^
		Sim (n=202)	Não (n=1.247)	Total (n=1.449)	
		n (%)	n (%)	n (%)	
Adulto jovem (<30 anos)	Sim	84 (12,8)	570 (87,2)	654 (45,1)	0,274
	Não	118 (14,8)	677 (85,2)	795 (54,9)	
Identidade de gênero	Trans	3 (6,1)	46 (93,9)	49 (3,4)	0,108
	Cis	199 (14,2)	1.201 (85,8)	1.400 (96,6)	
Orientação homossexual	Sim	178 (14,6)	1.044 (85,4)	1.222 (84,3)	0,111
	Não	24 (10,6)	203 (89,4)	227 (15,7)	
Situação conjugal	Sem um parceiro	151 (13,8)	943 (86,2)	1.094 (75,5)	0,761
	Com um parceiro	51 (14,4)	302 (85,6)	353 (24,4)	
Raça/cor da pele	Não branca	118 (13,6)	749 (86,4)	867 (59,8)	0,658
	Branca	84 (14,4)	498 (85,6)	582 (40,2)	
Possuir religião	Sim	106 (14,8)	611 (85,2)	717 (49,5)	0,254
	Não	91 (12,7)	625 (87,3)	716 (49,4)	
Ensino superior	Sim	177 (13,9)	1.099 (86,1)	1.276 (88,1)	0,836
	Não	25 (14,5)	148 (85,5)	173 (11,9)	
Renda formal	Sim	144 (13,6)	917 (86,4)	1.061 (73,2)	0,503
	Não	58 (14,9)	330 (85,1)	388 (26,8)	

^a^Diferenças foram consideradas estatisticamente significativas quando p-valor<0,005.

Entre os fatores psicossociais relacionados à experiência prévia com o vírus mpox, 14,6% (n=211) dos participantes relataram diagnóstico anterior de mpox, enquanto 25,5% (n=369) relataram que conhecem pessoas próximas que foram diagnosticadas com mpox. Ainda, 12,1% (n=175) informaram ter tido contato íntimo com pessoa diagnosticada com a mpox (p-valor<0,001),78,6% (n=1.139) afirmaram que divulgariam o estado sorológico da mpox, e a não divulgação esteve associada à maior vulnerabilidade (p-valor 0,095). Quanto às percepções sociais, o medo de discriminação foi relatado nos serviços de saúde (n=810; 55,9%), entre amigos (n=948; 65,4%) e entre familiares (n=743; 51,3%). Por fim, 84,1% (n=1.218) relataram a crença de que alguém pode sofrer homofobia por causa da mpox, e a ausência dessa crença mostrou associação com maior vulnerabilidade (p-valor 0,040) (Tabela 3).

**Tabela 3:** Análise bivariada dos fatores psicossociais associados aos comportamentos de vulnerabilidade à saúde entre homens que fazem sexo com homens no contexto da mpox. Brasil, 2022 (n=1.449)

Variáveis		Comportamentos de vulnerabilidade à saúde			p-valor^a^
		Sim (n=202)	Não (n=1.247)	Total (n=1.449)	
		n (%)	n (%)	n (%)	
Diagnóstico anterior de mpox	Sim	43 (20,4)	168 (79,6)	211 (14,6)	0,003
	Não	159 (12,8)	1.079 (87,2)	1.238 (85,4)	
Conhece pessoas próximas que foram diagnosticadas com a mpox	Sim	52 (14,1)	317 (85,9)	369 (25,5)	0,922
	Não	150 (13,9)	930 (86,1)	1.080 (74,5)	
Contato íntimo com uma pessoa diagnosticada com a mpox	Sim	41 (23,4)	134 (76,6)	175 (12,1)	<0,001
	Não	161 (12,6)	1.113 (87,4)	1.274 (87,9)	
Divulgaria o estado sorológico da mpox	Sim	150 (13,2)	989 (86,8)	1.139 (78,6)	0,095
	Não	52 (16,9)	256 (83,1)	308 (21,3)	
Medo de ser discriminado pelos serviços de saúde	Sim	123 (15,2)	687 (84,8)	810 (55,9)	0,142
	Não	79 (12,5)	554 (87,5)	633 (43,7)	
Medo de ser discriminado pelos amigos	Sim	133 (14,0)	815 (86,0)	948 (65,4)	0,893
	Não	69 (13,8)	432 (86,2)	501 (34,6)	
Medo de ser discriminado pela família	Sim	114 (15,3)	629 (84,7)	743 (51,3)	0,114
	Não	88 (12,5)	618 (87,5)	706 (48,7)	
Crença de que alguém pode sofrer homofobia por causa da mpox	Sim	159 (13,1)	1.059 (86,9)	1.218 (84,1)	0,022
	Não	43 (18,8)	186 (81,2)	229 (15,8)	

^a^Diferenças foram consideradas estatisticamente significativas quando p-valor<0,005.

Entre os comportamentos, 67,0% (n=971) relataram que o tipo de parceria sexual (últimos seis meses) era fixo+casual, e 99,9% (n=1.447) afirmaram que foram imunizados contra a covid-19. Quanto às mudanças após o surto, 55,1% (n=799) relataram que pararam de fazer sexo casual após a crise de mpox. Sobre testagem, 83,9% (n=1.216) relataram que seriam submetidos a testes de pré e de pós-exposição para a mpox (Tabela 4). 

**Tabela 4:** Análises bivariadas dos fatores comportamentais associados aos comportamentos de vulnerabilidade à saúde entre homens que fazem sexo com homens no contexto da mpox. Brasil, 2022 (n=1.449)

Variáveis		Comportamentos de vulnerabilidade à saúde			p-valor^a^
		Sim (n=202)	Não (n=1.247)	Total (n=1.449)	
		n (%)	n (%)	n (%)	
Tipo de parceria sexual (últimos seis meses)	Sem parceiro	10 (11,2)	79 (88,8)	89 (6,1)	0,419
	Fixo	59 (15,6)	318 (84,4)	377 (26,0)	
	Fixo + Casual	131 (13,5)	840 (86,5)	971 (67,0)	
Foi imunizado contra a covid-19	Sim	201 (13,9)	1.246 (86,1)	1.447 (99,9)	0,141
	Não	1 (50,0)	1 (50,0)	2 (0,1)	
Parou de fazer sexo casual após o surto de mpox	Sim	106 (13,3)	693 (86,7)	799 (55,1)	0,411
	Não	96 (14,8)	554 (85,2)	650 (44,9)	
Seria submetido a testes de pré e pós-exposição para a mpox	Sim	129 (10,6)	1.087 (89,4)	1.216 (83,9)	<0,001
	Não	42 (38,5)	67 (61,5)	109 (7,5)	
Prática sem preservativo	Sim	105 (13,0)	703 (87,0)	808 (55,8)	0,243
	Não	97 (15,1)	544 (84,9)	641 (44,2)	
Usa aplicativos para procurar sexo	Sim	112 (13,6)	709 (86,4)	821 (56,7)	0,707
	Não	90 (14,3)	538 (85,7)	628 (43,3)	
Usa saunas para procurar sexo	Sim	23 (10,6)	194 (89,4)	217 (15,0)	0,123
	Não	179 (14,5)	1.053 (85,5)	1.232 (85,0)	
Procura sexo em locais públicos (festas e clubes)	Sim	50 (11,0)	403 (89,0)	453 (31,3)	0,031
	Não	152 (15,3)	844 (84,7)	996 (68,7)	
Uso de *glory hole*	Sim	7 (7,4)	88 (92,6)	95 (6,6)	0,056
	Não	195 (14,4)	1.159 (85,6)	1.354 (93,4)	
Prática de chemsex	Sim	58 (20,6)	224 (79,4)	282 (19,5)	<0,001
	Não	144 (12,3)	1.023 (87,7)	1.167 (80,5)	
Prática de cruising	Sim	27 (14,3)	162 (85,7)	189 (13,0)	0,883
	Não	175 (13,9)	1.085 (86,1)	1.260 (87,0)	
Prática de bugchasing	Sim	32 (25,4)	94 (74,6)	126 (8,7)	<0,001
	Não	170 (12,8)	1.153 (87,2)	1.323 (91,3)	
Práticas sexuais desafiadoras	Sim	46 (20,5)	178 (79,5)	224 (15,5)	0,002
	Não	156 (12,7)	1.069 (87,3)	1.225 (84,5)	
Participação em orgias	Sim	24 (14,9)	137 (85,1)	161 (11,1)	0,707
	Não	178 (13,8)	1.110 (86,2)	1.288 (88,9)	

^a^Diferenças foram consideradas estatisticamente significativas quando p-valor<0,005.

No campo das práticas sexuais, 55,8% (n=808) relataram prática sem preservativo, 56,7% (n=821) que usam aplicativos para procurar sexo, 15,0% (n=217) que usam saunas para procurar sexo e 31,3% (n=453) que procuram sexo em locais públicos (festas e clubes). Sobre práticas específicas, o uso de *glory holes* (n=95; 6,6%) apresentou tendência de associação com menor prevalência do desfecho, embora sem significância estatística (p-valor 0,056). A prática de chemsex (n=282; 19,5%) apresentou associação significativa (p-valor<0,001), enquanto a prática de *cruising *(n=189; 13,0%) não mostrou significância estatística (p-valor 0,883). A prática de *bugchasing *(n=126; 8,7%) (p-valor<0,001) e as práticas sexuais desafiadoras (n=224; 15,5%) (p-valor 0,002) também se associaram ao desfecho. Já a participação em orgias (n=161; 11,1%) não apresentou significância estatística (p-valor 0,707) (Tabela 4).

O modelo multivariado incluiu uma variável de ajuste e identificou três variáveis associadas à maior prevalência de comportamentos de vulnerabilidade à saúde: ter contato íntimo com alguém diagnosticado com a mpox (RP ajustada 1,82; IC95% 1,33; 2,48); não divulgaria o estado sorológico de mpox (RP ajustada 1,40; IC95% 1,05; 1,87); e não acreditar que as pessoas possam sofrer homofobia por causa da mpox (RP ajustada 1,39; IC95% 1,01; 1,90). O uso de *glory holes *para sexo apresentou associação com prevalência 57% menor que o desfecho (RP ajustada 0,43; IC95% 0,20; 0,92) (Tabela 5).

**Tabela 5:** Razões de prevalência ajustadas (RP ajustada) e intervalos de confiança de 95% (IC95%) para fatores associados aos comportamentos de vulnerabilidade à saúde entre homens que fazem sexo com homens no contexto da mpox. Brasil, 2022 (n=1.449)

Variáveis	β^a^	RP ajustada (IC95%)	p-valor^b^
Intercept	-2,487	0,083 (0,04; 0,18)	<0,001
Ter contato íntimo com alguém diagnosticado com a mpox	0,596	1,82 (1,33; 2,48)	<0,001
Não divulgaria o estado sorológico de mpox	0,337	1,40 (1,05; 1,87)	0,023
Não acreditar que as pessoas possam sofrer homofobia por causa da mpox	0,328	1,39 (1,01; 1,90)	0,040
Uso de *glory holes* para sexo	-0,852	0,43 (0,20; 0,92)	0,029

^a^Coeficiente beta da regressão (β); ^b^Diferenças foram consideradas estatisticamente significativas quando p-valor<0,005.

## Discussão

Este estudo identificou que fatores sociais e comportamentais influenciaram fortemente a adoção de comportamentos de vulnerabilidade à saúde entre homens que fazem sexo com homens durante o surto de mpox no Brasil. Ter contato íntimo com pessoas diagnosticadas, omitir o status sorológico e não reconhecer a possibilidade de discriminação por homofobia estiveram associados à maior vulnerabilidade, enquanto o uso de estratégias alternativas de prática sexual, como *glory holes*, surgiu como fator protetivo. Esses achados evidenciaram que a percepção de risco e a forma como os indivíduos negociam sua intimidade têm impacto direto sobre atitudes preventivas de saúde.

O estudo apresentou algumas limitações. A amostra incluiu usuários de redes sociais/aplicativos de encontros, recrutados via *snowball *virtual, o que pode ter limitado pessoas com menor acesso digital e causado viés de seleção. A coleta de dados foi realizada de maneira autodeclarada e anônima, o que, apesar de garantir a confidencialidade e incentivar a participação, pode produzir vieses de memória e de desejabilidade social. O delineamento transversal pode impedir a definição de causalidade. A categorização da variável raça/cor da pele em “branco” e “não branco”, pelo baixo número de pessoas pretas e indígenas na amostra, pode ter restringido a análise interseccional. Essa restrição destaca a importância de pesquisas futuras com representatividade racial e abordagens analíticas mais amplas. A criação de desfecho composto também foi uma limitação, com reunião de comportamentos distintos com causas diversas. Ainda assim, o estudo forneceu evidências relevantes para compreender como desigualdades sociais e percepções de estigma modulam a vulnerabilidade à saúde.

No modelo multivariado, o contato íntimo com pessoa diagnosticada com a mpox foi fator mais fortemente associado. A proximidade afetiva pode reduzir a percepção de risco e levar a escolhas menos precavidas [19]. Deve ser considerada a possibilidade de negação ou de redução do risco entre indivíduos expostos ao vírus, o que indica ações de testagem e de monitoramento de contatos pós-exposição [20]. 

Em circunstâncias onde o vírus já se encontra no convívio imediato (indivíduo diagnosticado), a percepção de risco pode ser subavaliada, com adoção de atitudes menos precavidas [9], devido à falsa impressão de familiaridade e de confiança entre parceiros íntimos, o que gera menor adesão a medidas preventivas [21]. Em contextos de proximidade emocional/sexual, existe a propensão a ignorar precauções básicas, o que leva à necessidade de implementar estratégias educativas focadas em grupos próximos às pessoas diagnosticadas [21,22].

O sigilo do status sorológico para a mpox mostrou-se relevante ao ser associado a maior probabilidade de comportamentos de vulnerabilidade em saúde, possivelmente ligado ao medo de discriminação/estigma e da homofobia [23,24]. Esse silêncio, embora protetor no campo social, amplia a vulnerabilidade individual/coletiva e interrompe cadeias de transmissão [25]. Ações antiestigma para o compartilhamento do status sorológico sem retaliações sociais devem ser implementadas [26]. 

A descrença na possibilidade de homofobia associada à mpox associou-se à maior probabilidade de adesão a comportamentos de vulnerabilidade à saúde. Esse achado indicou que minimizar o impacto do preconceito pode levar a percepção de menor risco social e, consequentemente, a descuidos preventivos [26]. A negação do perigo de discriminação sugere afastamento da realidade de sofrer preconceito, o que se mostra alerta para a necessidade de campanhas educativas articuladoras nesse sentido (risco biológico/risco social). Reforça-se que a percepção do risco social (discriminação) impacta nas atitudes de saúde entre homens que fazem sexo com homens, o que chama a atenção ainda para campanhas de sensibilização mais abrangentes, não biologicistas, que envolvam consequências sociais da enfermidade e empatia [26,27]. 

Por outro lado, utilizar *glory holes* para sexo mostrou-se protetivo. Isso pode refletir percepção de redução do contato direto, mesmo sendo prática sexual que envolva riscos importantes de transmissão de infecções. Esse achado revelou a complexidade das estratégias de autoproteção, que podem ser simultaneamente interpretadas como protetivas e arriscadas [28]. A utilização de glory holes pode implicar em atividades sexuais arriscadas (contato com fluidos corporais), que representam risco considerável para a disseminação de infecções, como a mpox [28]. 

Houve predominância de comportamentos como sexo sem preservativo (bareback), uso de aplicativos para encontrar parceiros e existência de parceiros ocasionais/constantes. Essas atitudes podem ser identificadas como fatores de risco para a propagação de infecções sexualmente transmissíveis, o que foi analisado em estudos anteriores no Brasil [29]. Parte dos participantes, porém, relatou mudanças comportamentais: suspensão de encontros sexuais casuais após o surto de mpox, o que sinalizou espaço para ações educativas [20,29].

Diante da mpox, a vulnerabilidade à saúde entre homens que fazem sexo com homens é moldada por fatores sociais/comportamentais, do contato íntimo com pessoas diagnosticadas ao sigilo do status sorológico e à subestimação da homofobia, negação de estratégias alternativas de intimidade sexual que podem operar como proteção relativa. O enfrentamento dessa vulnerabilidade, para além de atitudes individuais do público afetado, requer integrar medidas biomédicas ao combate ao estigma, a ambientes seguros de cuidado, a políticas públicas equitativas, à escuta qualificada e à justiça social.

## Data Availability

O banco de dados e os códigos de análise utilizados na pesquisa podem ser solicitados ao autor correspondente.
